# Supplementary material for: Chromothripsis during telomere crisis is independent of NHEJ, and consistent with a replicative origin
Source: Genome Res. 2019 May;29(5):737–49. doi: 10.1101/gr.240705.118 (PMC6499312; doi:10.1101/gr.240705.118)
Supplement: Supplemental Material [file supp_gr.240705.118_Supplemental_file_1.zip › contigs/annotated_contigs/DB114/contig.2.DB114_length_515_mean_cov_7.28155339806.docx]

**DB114_length_515_mean_cov_7.28155339806**

```
AATTTGTAAAGCACTCCTTGCTGTGTAAAGCAGTTATCACAGTGCTTGGGCACATAGATTTCAATGAAAGCTAGTCAATATCATTCTTA
 >chrX:11736093-11736404 + E=1e-175 p=4e-02
GGATTTTCACGATTATTCTGCTTGAAACTTCAGCTTTGAGAATAAAGCAAACTTCATCAAAGGAGTATGTAGACATAGTTAATTTTCAC

GTGTTACTCCCAGTCAGGGTTTCTCAACCTTTGCAGGACTGAAATTTGGAACTGGACAATTCTTTATTGGGGGAATGACCTGTACATGG

TAGGATGTTGATCAATGTCACTGGTCTCTACTCATTAGAGTCT|A|TATGTTTTTGCTTTCCTTCACGATTAGTTTAAAATCATATTGG
                                            >chrX:11737096-11737301 + E=9e-112
AATTTAAAGGAGGATTTAGCAGAATATCAACTTCTGTGAGAAAATGATGAAAGGAAAAACAATTGGGTCTTTTGAATGAAACAGGTTGC

TGCGGGTGGCTATAACAAGTAAACCACTTAAGTTCAATGGCTAAATTAAACAAGACAGGAGCTTAATTCTCA
```
